# Large-scale fabrication of achiral plasmonic metamaterials with giant chiroptical response

**DOI:** 10.3762/bjnano.7.83

**Published:** 2016-06-24

**Authors:** Morten Slyngborg, Yao-Chung Tsao, Peter Fojan

**Affiliations:** 1 Department of Physics and Nanotechnology, Aalborg University, Skjernvej 4A, 9220 Aalborg East, Denmark

**Keywords:** biomolecule sensing, extrinsic chiral metamaterials, scalable fabrication

## Abstract

A variety of extrinsic chiral metamaterials were fabricated by a combination of self-ordering anodic oxidation of aluminum foil, nanoimprint lithography and glancing angle deposition. All of these techniques are scalable and pose a significant improvement to standard metamaterial fabrication techniques. Different interpore distances and glancing angle depositions enable the plasmonic resonance wavelength to be tunable in the range from UVA to IR. These extrinsic chiral metamaterials only exhibit significant chiroptical response at non-normal angles of incidence. This intrinsic property enables the probing of both enantoimeric structures on the same sample, by inverting the tilt of the sample relative to the normal angle. In biosensor applications this allows for more precise, cheap and commercialized devices. As a proof of concept two different molecules were used to probe the sensitivity of the metamaterials. These proved the applicability to sense proteins through non-specific adsorption on the metamaterial surface or through functionalized surfaces to increase the sensing sensitivity. Besides increasing the sensing sensitivity, these metamaterials may also be commercialized and find applications in surface-enhanced IR spectroscopy, terahertz generation and terahertz circular dichroism spectroscopy.

## Introduction

In recent years metamaterials have attracted a tremendous amount of attention owing to their unique properties enabling the fabrication and design of devices hitherto impossible. These properties have found implementations in various fields such as optics [1], improved photovoltaic devices [2], electronics [3], surface-enhanced infrared spectroscopy [4], Raman spectroscopy [5] and biosensors [6].

Planar chiral metamaterials (PCMs) have also attracted attention because of their negative refractive index [7–8] and optical activity [9] such as circular dichroism (CD) [10]. Among other things, this makes them useful for the production of sensing devices for organic molecules and biomolecules [10]. Recently, the phenomena of the long proposed extrinsic chirality dating back to 1945 [11] have been observed experimentally with materials that are achiral [12]. These extrinsic chiral metamaterials (ECMs) demonstrate CD responses that are orders of magnitudes larger than their PCM counterpart [12]. ECMs typically consist of achiral subwavelength hole arrays where the chiroptical response originates from a large area excitation of surface plasmon polariton (SPP) waves. Compared to the localized surface plasmon resonance from PCMs, SPP waves from ECMs are extremely sensitive to the angle of incidence and less sensitive to structural imperfections [13]. Furthermore, ECMs are defined by having a zero response angle, which is the angle where the ECMs exhibit mirror symmetry and hence do not yield a CD response. Though some PCMs show promise as they also yield huge CD responses through FANO resonance [14], the greatest advantage of ECMs is that sensing of biomolecules can be performed with only one sample in one experiment by inverting the tilt of the sample, whereas PCMs requires one samples of both enatiomeric structures and independent experiments with both samples.

To date, only very few different ECMs other than hole arrays [13,15–16] and the original suggested U-shaped and split ring structures [12], have been investigated. These have been thoroughly studied [17–20] and other structures including theoretical suggestions are limited to plasma sphere arrays [21], gold dot arrays [22], gold square arrays [23], metal disk arrays [24], two layer hole arrays [25], polystyrene sphere templates for gold deposition [26] and structures formed by carbon nanotubes [27].

However, in order to be able to apply these metamaterials in sensing devices of organic molecules and biomolecules a reliable large-area fabrication method is required. State-of-the-art fabrication techniques are based on electron beam lithography or focused ion beam milling, which both are expensive and time consuming methods. Large-scale fabrication of PCMs have been attempted to some degree applying different approaches such as glancing angle deposition [28], scaffold ornamentation [29–30], individual chiral nanoparticles [31], preassembled nanoparticles [32–34] and a variety of colloidal nanolithography techniques [35–37]. Compared to the above mentioned PCMs, the experimentally proven ECMs only comprise structures from polystyrene sphere templates for gold deposition [26], structures formed by carbon nanotubes [27] and larger U-shaped structures by nanoimprint lithography (NIL) [38] which have been scalably fabricated.

In the present work we present a novel route towards the large-scale fabrication of ECMs and metamaterials in general. These structures have never been reported before and add to the scarce amount of experimentally investigated ECMs. Our fabrication approach is based on a two-step thermal NIL process with subsequent glancing angle metal deposition. The master mold for the NIL was fabricated by anodic oxidation of an aluminum (Al) substrate, which has been demonstrated previously [39–40]. During this process a disordered honeycomb structure is formed in the substrate. By controlling different parameters the interpore distance has been varied. ECMs with different interpore distance were investigated with CD spectroscopy and scanning electron microscopy (SEM). Furthermore, by altering the interpore distance and the glancing angle for metal deposition it was possible to tune the obtained CD signals from UV all the way to IR wavelengths. As a proof of concept, the resonance shifts of SPPs were studied upon interactions between a protein or a chiral organic molecule and the ECM surface.

## Results and Discussion

**Figure 1:**
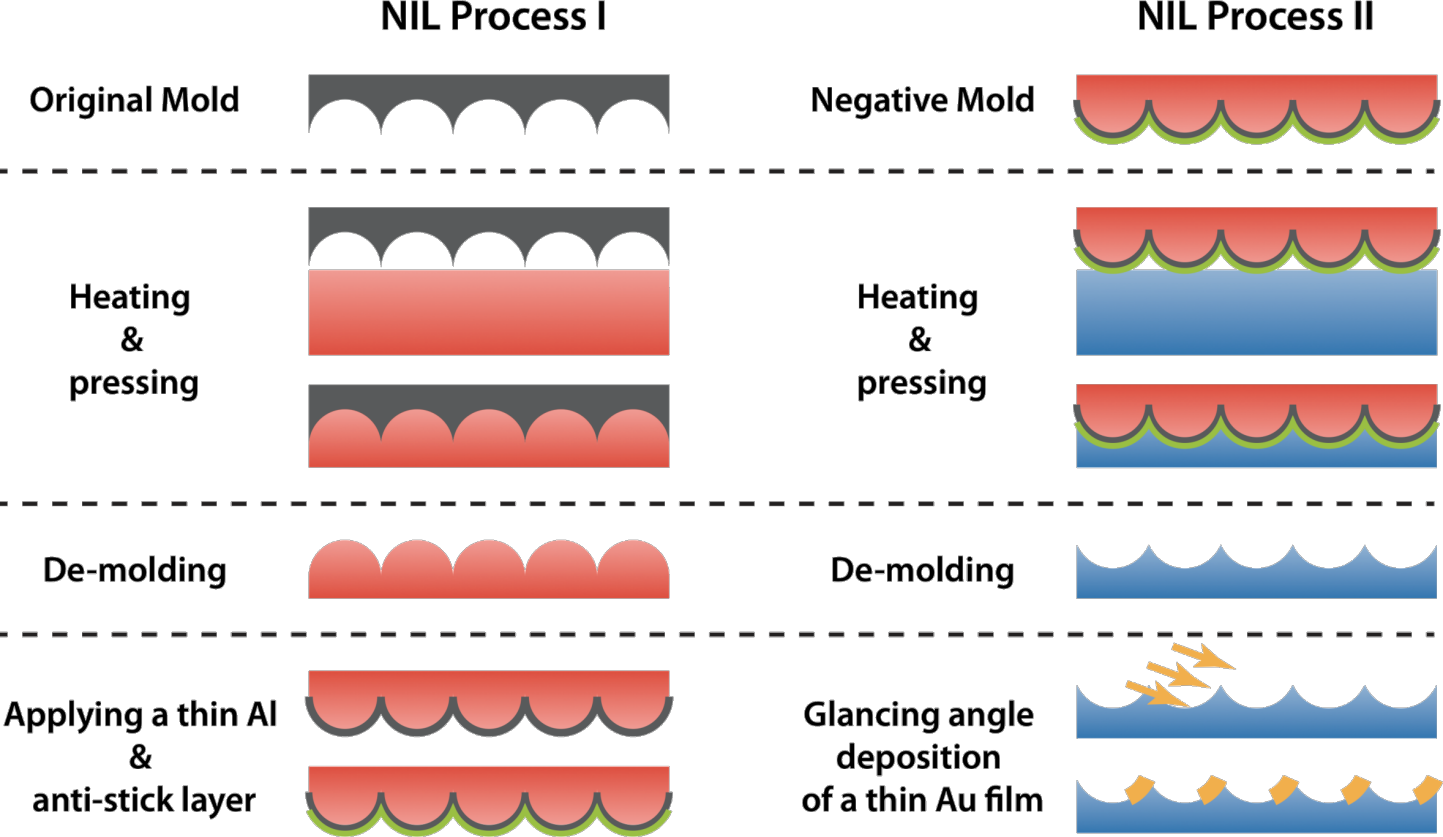
Schematical overview of the ECM fabrication process. First an original mold with a honeycomb structure is cast in a TOPAS polymer substrate. After de-molding a thin Al and anti-stick layer is applied to produce a negative mold. This negative mold is then cast in PMMA to produce the original honeycomb structure. This structure is used for initiation of glancing angle deposition of a 30 nm Au layer.

**Figure 2:**
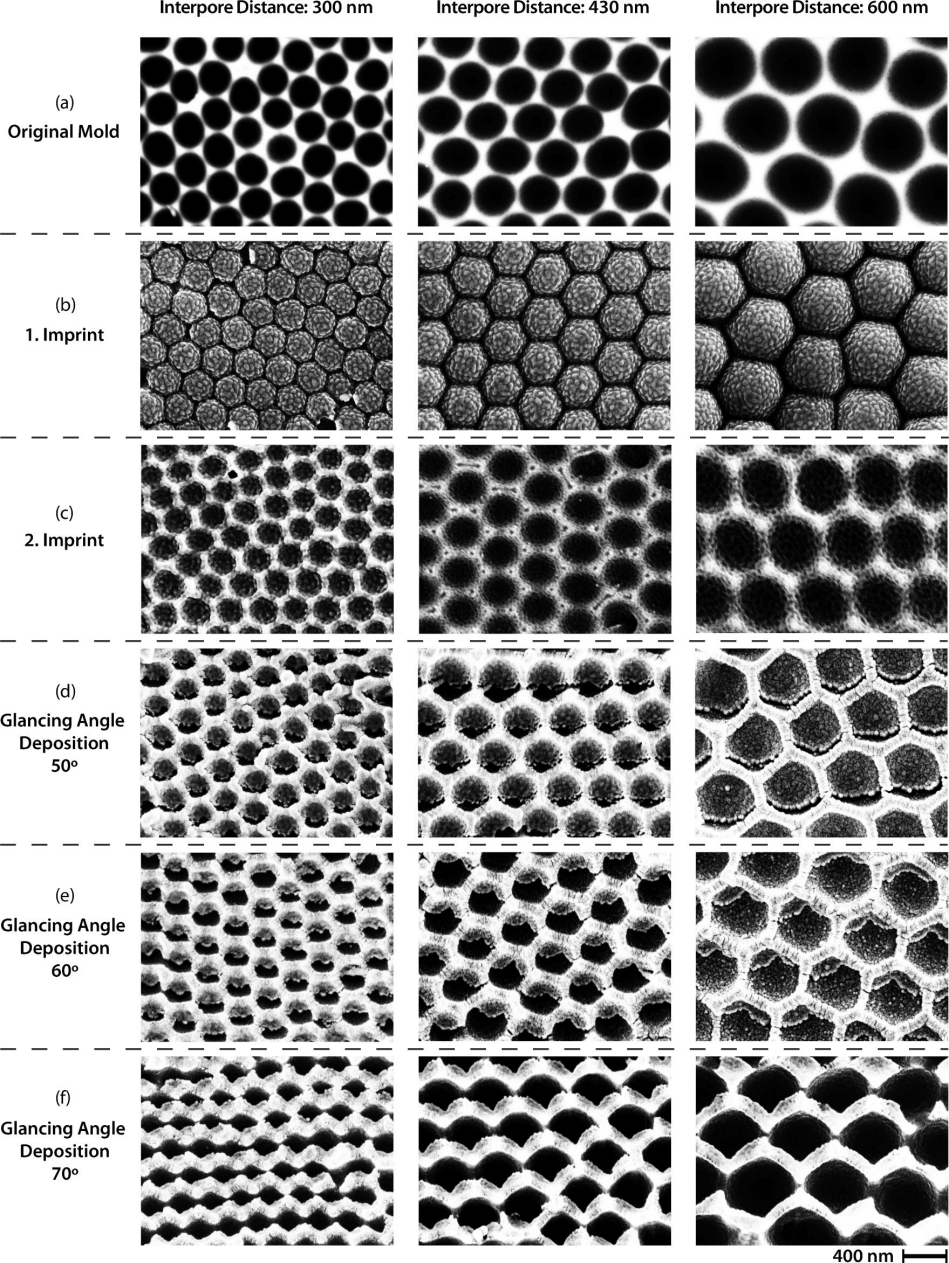
SEM pictures of (a) the original molds (b) 1. imprints with a thin Al layer (c) 2. imprints with a 2 nm Au layer and (d–f) all the different ECMs produced by glancing angle deposition of 30 nm Au at 50, 60 and 70°. The scale bar is shown in the bottom right corner.

The ECM fabrication process is illustrated schematically in Figure 1. The original mold was fabricated by a self-ordering anodic oxidation of Al foils as described in a previous study [39]. In this process pores are produced in the Al surface with a honeycomb structure. However, the pattern is not perfect and several types of defects are present, which are inevitably transferred to the final ECMs. By applying different acids and voltages in the anodic oxidation process, original molds with three different interpore distances (300, 430 and 600 nm) have been prepared as previously described [40]. These served as master molds in the following NIL process (Figure 2a).

The first imprints were cast in TOPAS polymer substrates. After de-molding the TOPAS structures were sputter-coated with a thin Al film and subsequently an anti-stick monolayer was applied to produce negative molds (Figure 2b). The negative molds were then cast in PMMA polymer substrates (Figure 2c). The honeycomb pattern in the PMMA substrates served as the starting structure of the glancing angle deposition of Au films. By varying the deposition angle (50, 60 and 70°), three different samples have been fabricated for each honeycomb interpore distance (Figure 2d–f).

**Figure 3:**
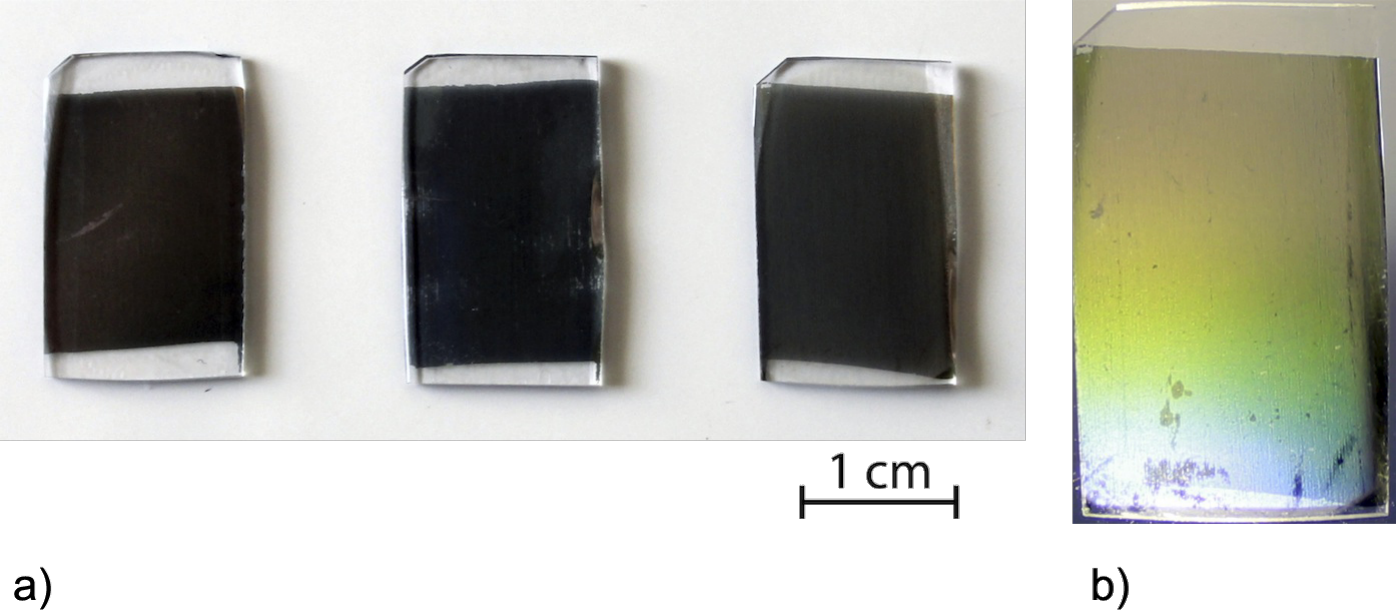
(a) Photography of the ECM samples prepared at a deposition angle of 60° and interpore distance of 300, 430 and 600 nm from the left, respectively. The sizes were chosen as to be compatible with commercial dismantled cuvettes and only a small area of the sample is probed. (b) Photography of the sample prepared by a deposition angle of 60° and interpore distance of 600 nm with flashlight to illustrate the diffraction pattern (the scale bar does not apply to this photo).

In total, nine different samples have been prepared, each covering approximately 3 cm^2^, together with three reference samples of a deposition angle of 0°. Figure 3a shows a photograph of the samples prepared at a deposition angle of 60°, the samples with 600 nm interpore distance show good diffraction (Figure 3b) while the samples with 430 nm interpore distance show moderate blue diffraction and the samples with 300 nm show no diffraction. However, the size of the samples are only limited by the size of the original molds, which were fabricated by another scalable technique, but the size of the samples was chosen to be compatible with commercial dismantled cuvettes and only a small area of the sample are probed at any given time.

### The criteria for extrinsic chiral metamaterials

**Figure 4:**
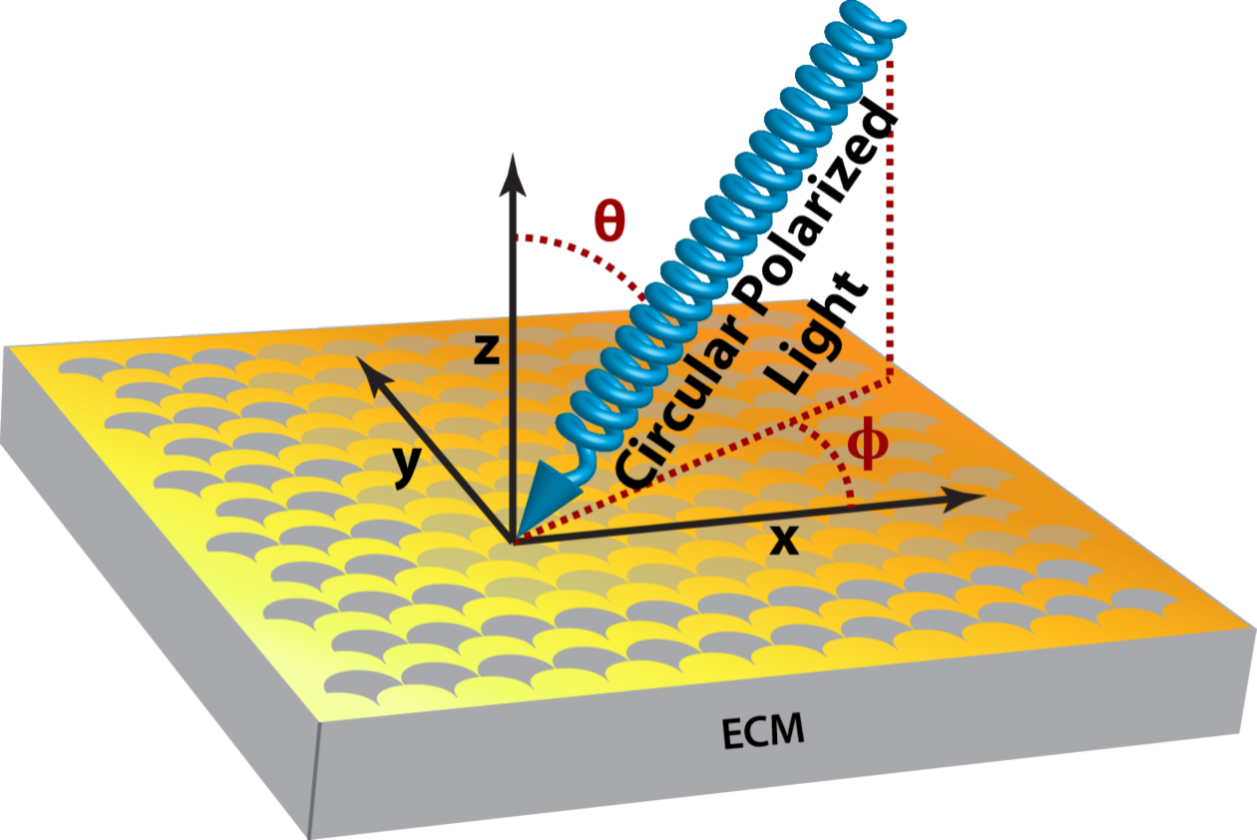
The two principle angles, θ and 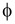, of incident light with respect to the ECM structures.

A chiroptical response is only obtained from structures lacking mirror symmetry. As the unit structures of ECMs are not natively chiral, they rely greatly on the orientation of the sample with respect to the direction of the incident light causing a broken mirror symmetry. By careful design it is possible to design the ECM structure in such a way that no CD response is observed when the face of the ECM substrates are oriented perpendicular to the incident light. Hence a CD signal is only obtained when the sample is tilted out of one of the two angles θ and 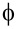 (Figure 4).

**Figure 5:**
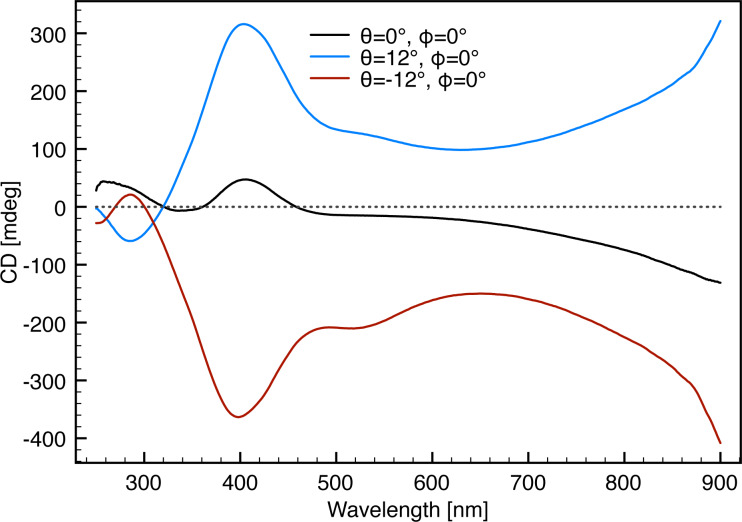
The resulting CD response from three ECM orientations (θ = 0°, 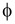 = 0°), (θ = 12°, 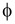 = 0°) and (θ = −12°, 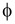 = 0°) of the ECM with 300 nm interpore distance and 60° deposition angle. It is evident that (θ = 0°, 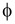 = 0°) shows no significant response, while the two other orientations yield inverted line shapes.

This enables the investigation of the enantiomeric ECM structures (right-handed and left-handed structure) to be probed in the same experiment, by inverting the tilt of the ECM with respect to the normal ECM face angle. The investigated ECMs have been designed in a way that it was possible to invert the CD signal by tilting the samples with respect to the θ angle while retaining a 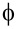 angle of 0° (Figure 5). The observed response from the bare sample when irradiated with light at a normal incident angle may have three different causes: 1) various structural imperfections, 2) a spread in structure size, 3) the 3D nature of the ECM structure causing some intrinsic chirality. However, this CD response at 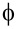 = 0° has also been observed by ECMs produced with focused ion beam milling [13].

The ECM property that allows for the measurement of the enantiomeric structures from one sample, yields several advantages over PCMs in biosensor applications: 1) PCMs require fabrication of the two enantiomeric samples, which increases the cost and the risk of imperfections. 2) With the use of PCMs it is necessary to perform two independent experiments, which is time-consuming and difficult to implement into a commercial product. 3) Since the CD response is concentration-dependent, two independent PCM experiments are troublesome to interpret. All of these disadvantages with PCMs are totally avoided by the use of ECMs, which are both cheaper, more reliable and only require one experiment in biosensor applications.

### Probing the zero-response angle

**Figure 6:**
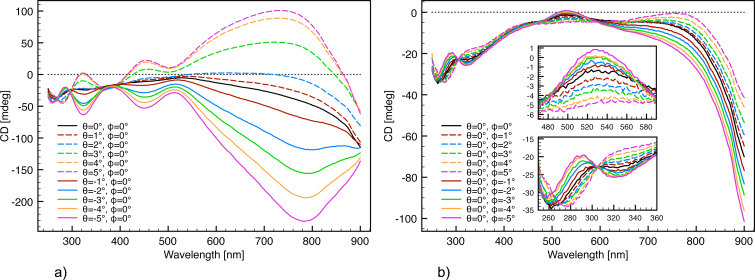
Angular scans and corresponding CD response from the ECM fabricated with 430 nm interpore distance and 50° glancing angle deposition. (a) Independent scan of the θ angle defined in Figure 4. (b) Independent scan of the 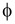 angle defined in Figure 4.

The zero-response angle of the ECM with 450 nm interpore distance and 50° glancing angle deposition was identified by scanning the θ and 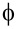 angles separately (Figure 6). This ECM substrate was chosen as it exhibits more and stronger resonance modes than the other samples and will be used in most subsequent experiments. While the CD response was extremely sensitive towards angular rotation about the θ angle plane (Figure 6a), it exhibited a lower angular dependence on the 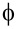 angle plane (Figure 6b).

Furthermore, it is evident from the angular scans (Figure 6) that the ECM has an intrinsic left-hand chirality. Hence, the zero-response angle is not identified by the lowest CD response but as the center of the enantiomeric signals. From the θ angle scan (Figure 6a), it appears as if θ = −1° and θ = +2° display the least response. Hence, the θ angle resulting in minimum extrinsic chirality is between 0° and +1°. As virtually no difference in line shape is observed between θ = 0° and θ = +1°, θ = 0° was used as zero-θ-angle throughout the paper for the sake of convenience.

**Figure 7:**
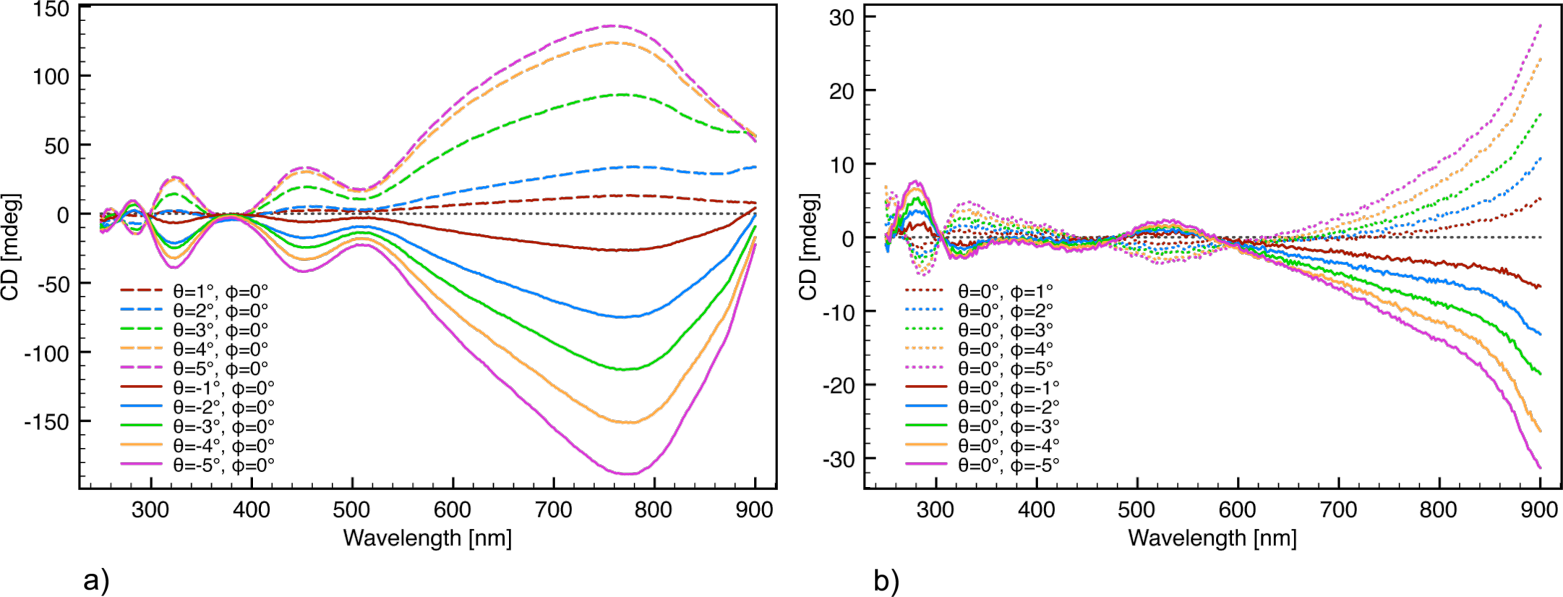
Angular scans with the intrinsic chirality subtracted. A much higher symmetrical CD response from the ECM enantiomers is observed compared to the angular scans in Figure 6. (a) Independent scan of the θ angle. (b) Independent scan of the 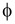 angle.

The two resonance modes located in the wavelength range of 260–360 nm of the 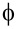 angle scan (Figure 6b) indicate that 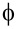 = 0° to 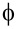 = −3° exhibits no extrinsic chirality. As virtually no difference in line shape is observed between these angles, 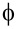 = 0° was used as zero-response angle. Applying θ = 0° and 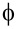 = 0° as reference results in a more symmetrical angular scan (Figure 7) indicating the precision of the determination of the zero-response angle.

### Influence of interpore distance and glancing angle deposition

**Figure 8:**
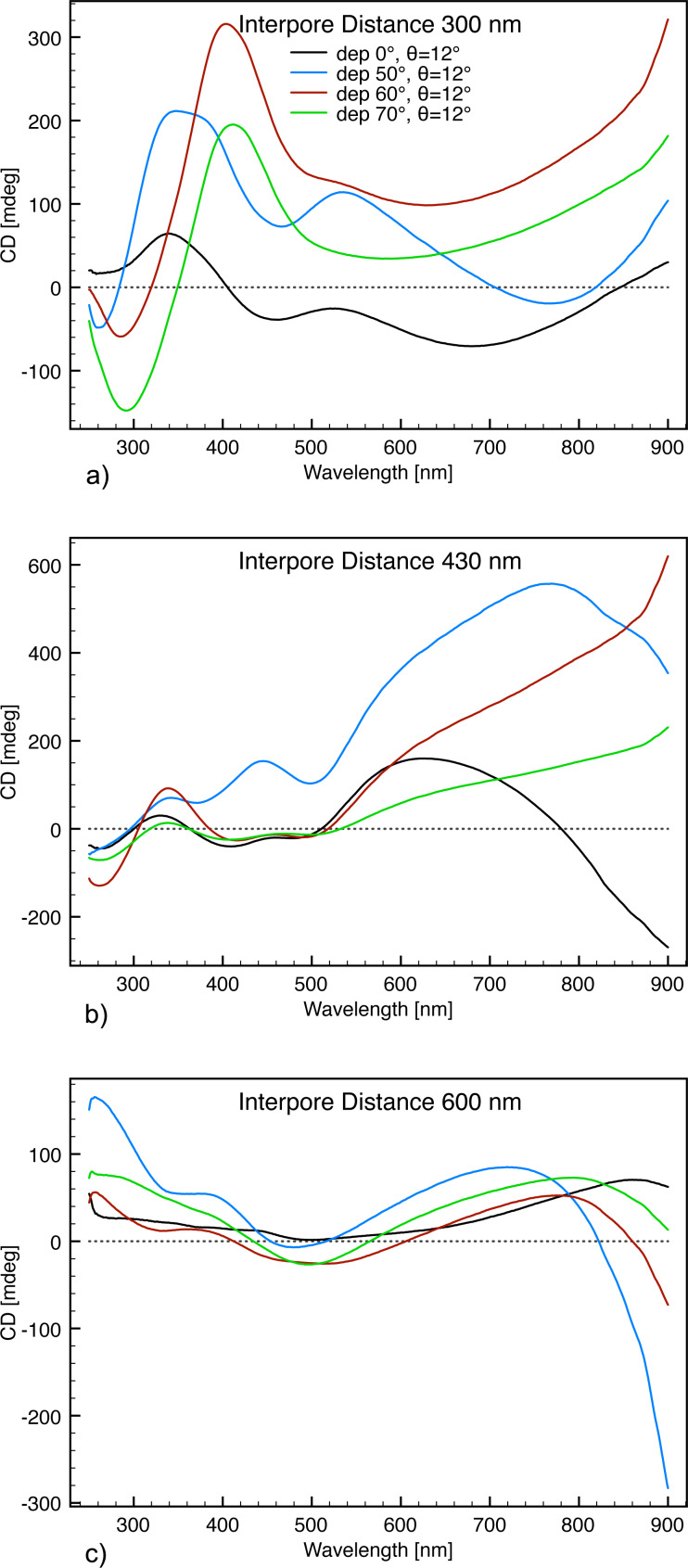
CD response from all the produced ECMs. (a) The CD response from the structures with 300 nm interpore distances. (b) The response from the structures with 430 nm interpore distances. (c) The response from the structures with 600 nm interpore distances. All measurements were recorded with an ECM orientation of θ = 12° and 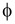 = 0°.

Structures with interpore distances of 300, 430 and 600 nm exhibited main plasmonic resonance ranges of about 300–550 nm, 450–800 nm and above 900 nm (Figure 8).

Compared to the relatively narrow linewidth of the CD response from ECMs comprised of hole arrays, the honeycomb ECMs exhibit a rather broad signal [13]. This is presumably related to the heterogeneity and 3D nature of the ECM structures, which is less dominant in the hole arrays fabricated by focused ion beam lithography. In spite of this, the CD linewidths of the present ECMs are comparable to those of gammadion PCMs, which have been previously used for biosensor applications [10]. Furthermore, compared to PCMs fabricated by another scalable technique [29], the CD linewidths of the honeycomb ECMs are more narrow. Hence the heterogeneity and 3D structure has not limited the use of the honeycomb ECMs for biosensing applications.

While the interpore distance of the ECM array is the main factor in the position of the SPP resonance, the angle of the Au deposition has mainly an influence on the line shape and the number of resonance peaks (Figure 8). The samples prepared at 0 and 50° glancing angle deposition show a similar line shape, as well as the samples prepared at 60 and 70°. This is reasonable as the angle deposition sensitivity, which determines the dependence of the resulting structure on the deposition angle, is very low at small angles but increases with large angles. The reason for this is found in the shadowing effect of the honeycomb structure. The glancing angle deposition is self-perpetuating at larger angles while small angles result only in minor shadowing effects. The samples prepared at 0 and 50°, in general, exhibit three to four distinct resonance peaks while the samples prepared at 60 and 70° only exhibit two to three distinct resonance peaks. This might be explained by the gold inside the hole arrays produced at 0 and 50° glancing angle deposition, resulting in a more complex 3D structure (Figure 2) and an additional origin of plasmonic resonance modes. The samples with 600 nm interpore distances had a main resonance wavelength above 900 nm (data not shown). These structures may find applications in other areas such as surface enhanced IR spectroscopy [41], terahertz generation [42] or THz-CD spectroscopy [43].

### Influence of the scanning angle and of intrinsic spatial structural variations

**Figure 9:**
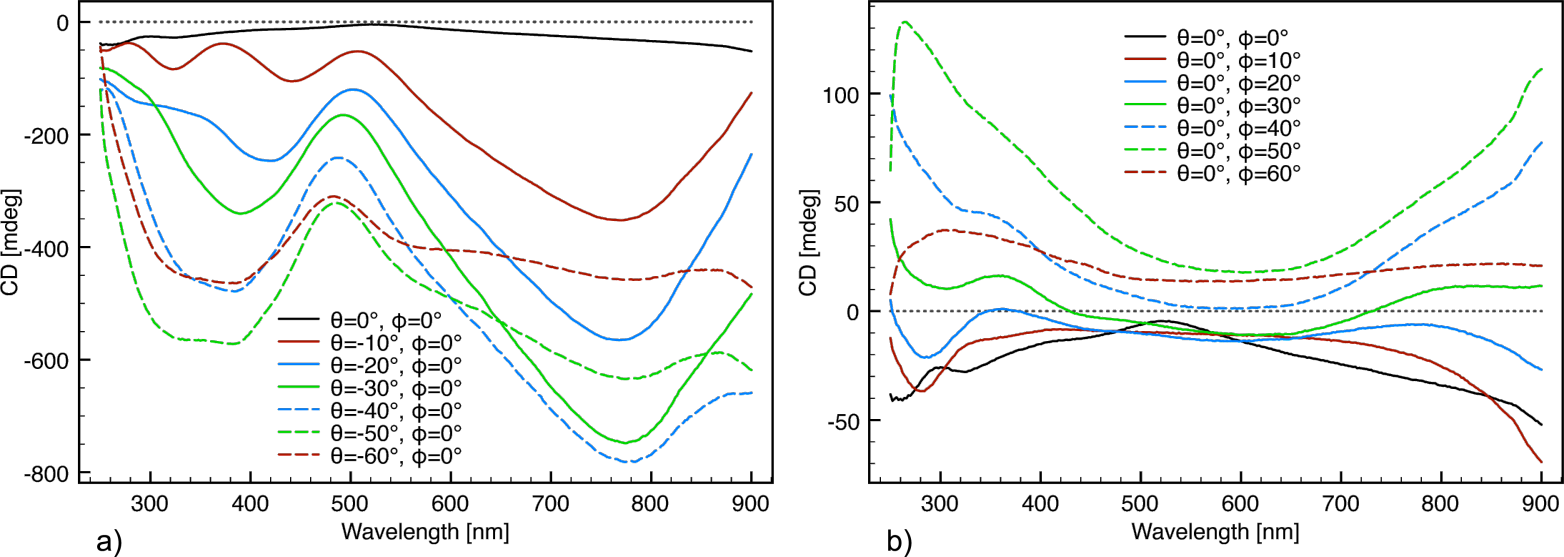
Angular scans with large angle increments and corresponding the CD response from the ECM fabricated with 430 nm interpore distance and 50° glancing angle deposition. (a) Independent scan of the θ angle. (b) Independent scan of the 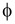 angle.

As mentioned above, the plasmonic resonance wavelength and intensity depends on the orientation of the ECMs. Upon rotating the sample in the θ angle plane (Figure 9a) it is evident that the two distinct resonance modes in the near-UV region merge upon increasing the θ angle from 10 to 20°. At θ = 30° this peak is blue-shifted. When the θ angle is increased to 40 and 50° the peak re-separates into two peaks. These changes may originate from the transition from a hole array towards a film with gratings upon increasing angles and subsequent larger backscattering. At θ = 60° the backscattering has increased significantly, resulting in a pronounced drop in CD response. Furthermore, the 770 nm plasmonic resonance mode does not show any angular dependence and remains located at the same wavelength.

From the 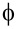 angle scan (Figure 9b) it is evident that only plasmonic resonance modes in the infrared and UV range are induced. Upon increasing the 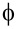 angle, the resonance modes in the UV range intensifies and changes of the resonance wavelengths occur. It is also noteworthy for other applications of the ECM, that a very strong resonance mode in the range of 900–1100 nm has been identified in other measurements (data not shown) but is outside of the measured range presented here as it is not relevant to protein sensing using CD spectroscopy. Similar to the θ angular scan, the CD response at a 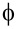 = 60° drops as a consequence of significantly increased backscattering.

**Figure 10:**
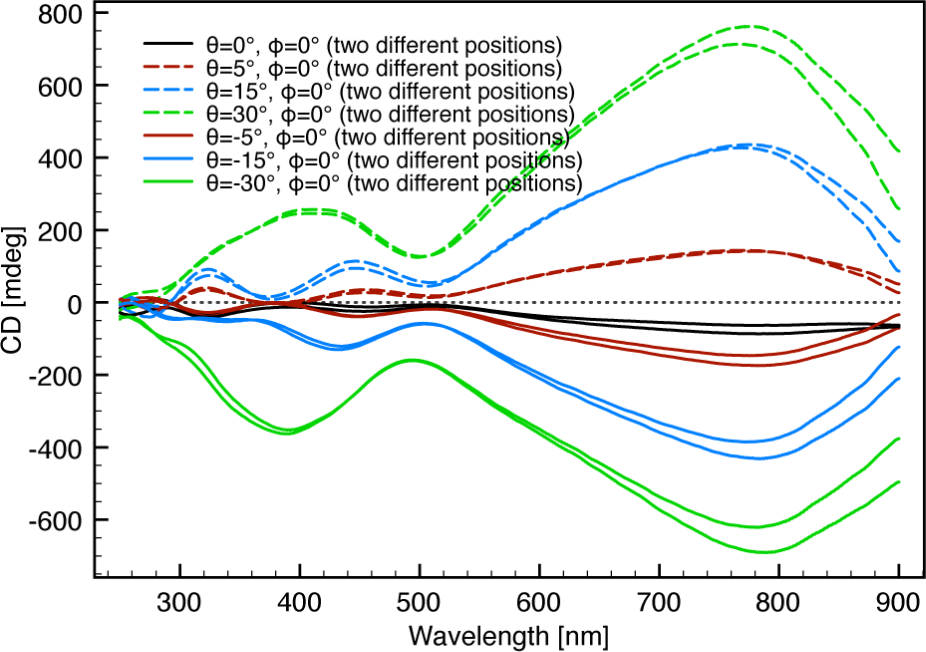
CD response from the ECM fabricated with 430 nm interpore distance and 50° glancing angle deposition in four different angle orientation and measured in two different positions 6 mm apart on the sample.

Typically, metamaterials are fabricated with a significantly smaller surface area than the beam profile used to probe the sample. However, the present ECMs are significantly larger, but with various structural imperfections. To validate the integrity of the sample fabricated with 430 nm interpore distance and a glancing angle deposition of 50° was probed in two different positions over 6 mm apart. Minor differences were observed (Figure 10), indicating small structural differences.

These differences are no larger than what has been reported for PCMs fabricated with e-beam lithography [10] or ECMs fabricated with ion beam milling [13] and most likely originate from structural imperfections. Another contributing factor might be that the ECM surface is much larger than the beam profile, hence the structures in the circumference of the beam profile may appear different from those substructures being completely probed. Beam profile induced imperfections are circumvented in usual fabrication techniques as the achievable sample area is much smaller than the beam profile.

### Sensing of chiral molecules

As a proof of concept, the sensing power of the honeycomb ECMs were investigated by monitoring the resonance shift upon adsorption of the chiral organic molecule cysteamine and the protein cytochrome c. cytochrome c acts as an electron shuttle and as a respiratory redox protein [44]. It also assists as an important mediator in the apoptotic pathways [44]. Due to a free surface-accessible cysteine it is well known to readily adsorb onto gold surfaces and is often used to study electron transfer in cyclic voltammetry [45]. Cysteamine is the simplest stable aminothiol, hence it readily adsorbs onto gold surfaces and forms a self-assembled monolayer which is often used as the first layer in the functionalization of surfaces [46].

**Figure 11:**
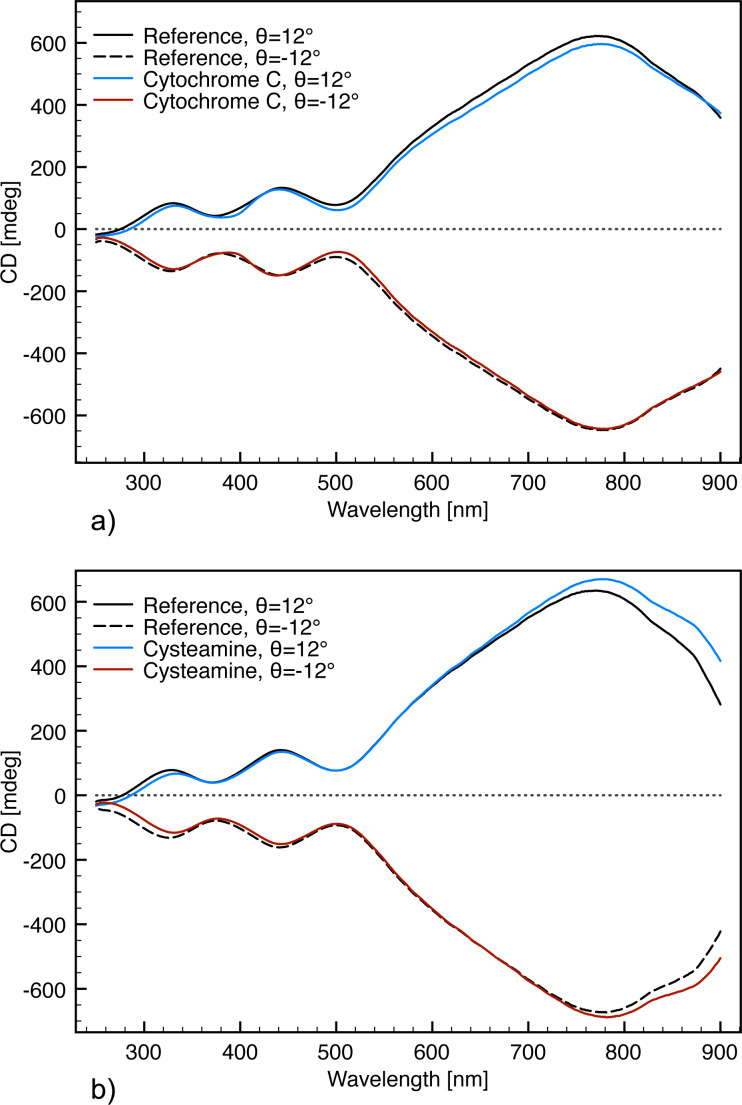
CD response from a bare ECM and with a molecule adsorbed on the surface. (a) CD response with and without cytochrome c adsorbed on the ECM surface. (b) CD response with and without Cysteamine adsorbed on the ECM surface. All measurements were recorded with an ECM orientation of θ = 12°; 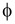 = 0° or θ = −12°; 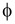 = 0°.

In all experiments the ECM fabricated with 430 nm interpore distance and a glancing deposition angle of 50° was used as it displays a well-defined response at ca. 770 nm compared to the other structures, together with two minor distinct peaks at ca. 440 nm and ca. 330 nm. The resulting spectra are presented in Figure 11 and the corresponding shifts compared to the bare structure are summarized in Table 1.

**Table 1:** Excitation shifts originating from different molecules.

molecule	Δλ_770 nm_ (θ_12_/θ_−12_)	Δλ_440 nm_ (θ_12_/θ_−12_)	Δλ_330 nm_ (θ_12_/θ_−12_)

cytochrome c	2/2 nm	−4/−4 nm	2/3 nm
cysteamine	7/12 nm	2/2 nm	4/5 nm

**Figure 12:**
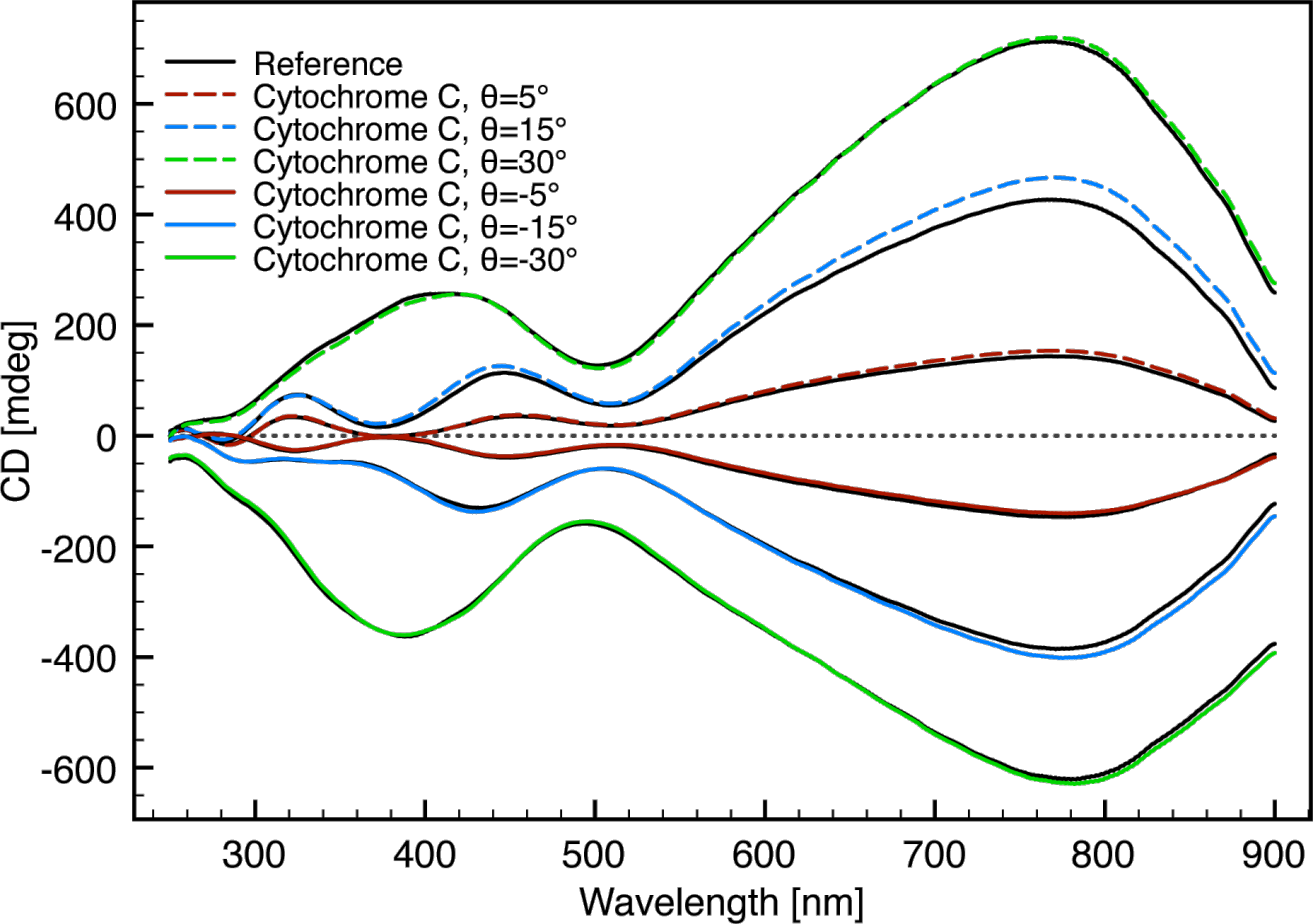
CD response from the ECM fabricated with 430 nm interpore distance and 50° glancing angle deposition with different angle orientation and measured with and without cytochrome c adsorbed on the surface.

A similar plasmonic resonance shift of the resonance mode around 770 nm upon adsorption of cytochrome c was observed at different θ angles (Figure 12 and Table 2). This indicates that the sensitivity of the plasmonic resonance towards changes in the local refractive index near the ECM surface is not effected by the θ angle. Only the resonance mode around 770 nm was used in this analysis as the resonances of the other modes are altered as described above and as a result are incomparable.

**Table 2:** Excitation shifts and dissymmetry factors of the plasmonic resonance at 770 nm at different θ angles, originating from adsorption of cytochrome c on the ECM fabricated with 430 nm interpore distance and a glancing angle deposition of 50°.

angle	Δλ_770 nm_	ΔΔλ_770 nm_

(θ_5_/θ_−5_)	1.4/1.5 nm	0.1 nm
(θ_15_/θ_−15_)	2.3/2.6 nm	0.3 nm
(θ_30_/θ_−30_)	2.2/2.4 nm	0.2 nm

It has been demonstrated that the dissymmetry factor [10] (ΔΔλ) of the shifts of the right-handed spectrum compared to the left-handed spectrum (ΔΔλ = Δλ_right_ − Δλ_left_) is an indication of an anisotropic adsorption (Table 3) [10]. Whereas a perfect isotropic adsorption would result in a ΔΔλ value of zero. Interestingly, the adsorption of the two molecules does not result in the same behavior in signal shifts. While the adsorption of cytochrome c did not result in a measurable dissymmetry shift, the adsorption of cysteamine caused a relatively large dissymmetry shift as indicated by the 770 nm signal. This is coherent with other studies, demonstrating that cysteamine forms a well-ordered layer on Au surfaces [47–48]. Furthermore, it has been reported that cytochrome c either adsorbs with the α-helix structure parallel to the Au surface [49] or that the protein instantly denaturates upon adsorption to the Au surface [50]. In either case this will result in an isotropic layer with respect to the incoming light, which is coherent with the obtained results.

**Table 3:** Dissymmetry factors, calculated from the shifts given in Table 1.

molecule	ΔΔλ_770 nm_	ΔΔλ_440 nm_	ΔΔλ_330 nm_

cytochrome c	0 nm	0 nm	−1 nm
cysteamine	−5 nm	0 nm	−1 nm

The average extent of the two plasmonic enantiomer shifts (Δλ_av_ = (Δλ_right_ + Δλ*_left_*)/2 gives an indication of the amount of molecules adsorbed on the gold surface of the metamaterial [10]. Instead of using the peak at 550 nm to determine the surface coverage as previously reported by PCMs [10], the peak at 330 nm has been used in this paper, since it is the peak located closest to the UV range (Table 4).

**Table 4:** Average shifts calculated from the shifts given in Table 1.

molecule	Δλ_AV,330nm_

cytochrome c	2.5 nm
cysteamine	4.5 nm

From the data it is apparent that both cytochrome c and cysteamine do interact with the ECM gold surface. Compared to another study using PCMs, it is apparent that the Δλ_AV,330nm_ of cytochrome c and cysteamine is comparable to the Δλ_AV,≈ 550nm_ of the proteins resulting from the highest adsorption on a PCMs gold surface [10]. As the sensitivity of the ECM resonance mode at 330 nm is unknown the exact surface coverage cannot be determined. However, the sensitivity is not expected to deviate significantly from that observed with PCMs at 550 nm. Based on this both cytochrome c and cysteamine are adsorbed in picogram quantities on the ECM Au surface.

The current results demonstrate that proteins and chiral organic molecules in general readily adsorb onto the ECM surface. This demonstrates that the ECMs may be used for detection of proteins and chiral organic molecules in a label-free way. Furthermore, the ease of cysteamine adsorption on the ECM surface suggests that other molecules such as ethanedithiol may be used to functionalize the ECM surface and provide the possibility to fabricate very sensitive sensor arrays.

## Conclusion

In conclusion, a strong chiroptical response has been demonstrated from different achiral plasmonic hole arrays. The arrays were fabricated by a scaleable technique while retaining control and order of the resulting arrays. This signifies a substantial improvement to standard fabrication methods such as focused ion beam and electron beam lithography concerning cost and production time. Furthermore, the use of a small chiral organic molecule and a protein has been used as a proof of concept for the sensing of biological and chiral organic molecules in picogram quantities by CD spectroscopy. The response of the presented ECMs was tunable within the UVA and IR regions, depending mainly on the interpore distance of the hole arrays. The deposition angle had an effect on the amount of resonance modes. Small glancing angle deposition resulted in complex ECM geometries and consequently more resonance modes were observed. The number of modes is larger than that which has been previously reported for PCMs [10,32,34]. This is a significant improvement, as more signals yield a more detailed protein fingerprint.

The enantiomeric form of the investigated ECMs were recorded by inverting the tilt of the same sample about the θ angle plane, effectively eliminating the disadvantages of PCMs having to fabricate two independent samples and conducting two separate experiments to probe the protein fingerprint. CD spectroscopy revealed that both cytochrome c and cysteamine readily adsorbed on the ECM gold surface, amounting to picogram quantities. Furthermore, the formation of a cysteamine layer on the ECM gold surface suggests that similar organic molecules may be used to fabricate functionalized surfaces applicable for sensors with increased sensitivity or arrays hereof in a cheap and scaleable way.

## Experimental

### Fabrication of extrinsic chiral metamaterials

The original molds were prepared by anodic aluminum oxidation using a custom-built anodization and wet-etching system. Al foils (99.98%, Advent Research Materials Ltd. AL103310) were used as substrates after cleaning in an ultrasonic bath with a sequence of acetone, deionized water and methanol for 1 min each. In total three types of molds were prepared with different interpore distances: 300, 430 and 600 nm. The substrate with 300 nm interpore distance was prepared by anodization in 0.3 M oxalic acid solution at 140 V and with a solution temperature of 283 ± 0.5 K for 40 min. The substrate with 430 nm interpore distance was prepared by anodization in 1 M phosphoric acid solution at 180 V and with a solution temperature of 273 ± 0.5 K for 100 min. The substrate with 600 nm interpore distance was prepared by anodization in 2 M citric acid solution at 285 V and with a solution temperature of 293 ± 0.5 K for 20 min. More details on the fabrication of the original mold has been reported previously [39]. The original molds were used to make negative imprints by thermal nanoimprint lithography using the EVG520HE semi-automated hot embossing system. This was done in TOPAS 5013L-10 substrates under vacuum with a stamping pressure of 1.25 bar and at 160 °C using the original molds. Next a 30 nm film was sputter-coated on the surface of the negative imprints. A monolayer of trichloro(1*H*,1*H*,2*H*,2*H*-perfluorooctyl)silane (25 vol % in toluene) was applied to the Al films by gas phase deposition for 1 h in a vacuum desiccator. These samples served as new molds for the second imprint in PMMA. The parameters of this imprint were similar to the once described above but were prepared at 120 °C. The negative molds were used several times without observable deterioration. The final ECMs were achieved by glancing angle deposition of 2 nm Cr followed by 50 nm Au at different deposition angles.

### Scanning electron microscopy

SEM measurements of the ECM surfaces were done in high vacuum (1·10^−6^ mbar) and an accelerating voltage 10 kV using a Zeiss 1540XB system and standard procedure. The samples were coated by 2 nm gold as to prevent a buildup of static charge.

### CD spectroscopy measurements

The ECMs were compatible with commercial available liquid CD cells with a path length of 0.1 mm and a total volume of 50 μL. The CD spectra were collected in normal incident mode where the samples were parallel to the detector and in tilted configuration, where the samples were tilted by 12°. CD spectra were collected using a commercial JASCO J-750 spectropolarimeter.

### Adsorption of cytochrome c and cysteamine onto extrinsic chiral materials

Cytochrome c was adsorbed on the substrates by incubation of 50 μL protein solution (1 mg·mL^−1^) for 1 h. Cysteamine was adsorbed by incubating 50 μL of solution (10 mM) for 24 h. The cysteamine solution was prepared with degassed Milli-Q water and the cytochrome c solution was prepared using 5 mM PBS buffer at pH 7.4.

### Surface regeneration of extrinsic chiral materials

ECMs were reused several times and before each experiment the substrates were submerged for 2 h in a sodium dodecyl sulfate solution, followed by a 30 min bath in a Hellmanex III solution at 37 °C. Finally, the substrates were cleaned in an oxygen-plasma cleaning unit for 1 h. After each step the ECMs were rinsed with Milli-Q water.

## References

[1] Valentine J, Zhang S, Zentgraf T, Ulin-Avila E, Genov D A, Bartal G, Zhang X (2008). Nature.

[2] Atwater H A, Polman A (2010). Nat Mater.

[3] Zia R, Schuller J A, Chandran A, Brongersma M L (2006). Mater Today.

[4] Abb M, Wang Y, Papasimakis N, de Groot C H, Muskens O L (2014). Nano Lett.

[5] Kühler P, Weber M, Lohmüller T (2014). ACS Appl Mater Interfaces.

[6] Yanik A A, Huang M, Kamohara O, Artar A, Geisbert T W, Connor J H, Altug H (2010). Nano Lett.

[7] Pendry J B (2004). Science.

[8] Zhang S, Park Y-S, Li J, Lu X, Zhang W, Zhang X (2009). Phys Rev Lett.

[9] Hannam K, Powell D A, Shadrivov I V, Kivshar Y S (2014). Phys Rev B.

[10] Hendry E, Carpy T, Johnston J, Popland M, Mikhaylovskiy R V, Lapthorn A J, Kelly S M, Barron L D, Gadegaard N, Kadodwala M (2010). Nat Nanotechnol.

[11] Bunn C (1945). Chemical Crystallography.

[12] Plum E, Liu X-X, Fedotov V A, Chen Y, Tsai D P, Zheludev N I (2009). Phys Rev Lett.

[13] Maoz B M, Ben Moshe A, Vestler D, Bar-Elli O, Markovich G (2012). Nano Lett.

[14] Zu S, Bao Y, Fang Z (2016). Nanoscale.

[15] Zambrana-Puyalto X, Vidal X, Molina-Terriza G (2014). Nat Commun.

[16] Arteaga O, Maoz B M, Nichols S, Markovich G, Kahr B (2014). Opt Express.

[17] Feng C, Wang Z B, Lee S, Jiao J, Li L (2012). Opt Commun.

[18] Shi J H, Zhu Z, Ma H F, Jiang W X, Cui T J (2012). J Appl Phys.

[19] Wang F, Wang Z, Shi J (2014). J Appl Phys.

[20] Sersic I, van de Haar M A, Arango F B, Koenderink A F (2012). Phys Rev Lett.

[21] Yannopapas V (2009). Opt Lett.

[22] Shaltout A, Liu J, Shalaev V M, Kildishev A V (2014). Nano Lett.

[23] Hashiyada S, Narushima T, Okamoto H (2014). J Phys Chem C.

[24] Kruk S S, Helgert C, Decker M, Staude I, Menzel C, Etrich C, Rockstuhl C, Jagadish C, Pertsch T, Neshev D N (2013). Phys Rev B.

[25] Boutria M, Oussaid R, Van Labeke D, Baida F I (2012). Phys Rev B.

[26] Belardini A, Benedetti A, Centini M, Leahu G, Mura F, Sennato S, Sibilia C, Robbiano V, Giordano M C, Martella C (2014). Adv Opt Mater.

[27] Yokoyama A, Yoshida M, Ishii A, Kato Y K (2014). Phys Rev X.

[28] Singh J H, Nair G, Ghosh A, Ghosh A (2013). Nanoscale.

[29] Yeom B, Zhang H, Zhang H, Park J I, Kim K, Govorov A O, Kotov N A (2013). Nano Lett.

[30] Schreiber R, Do J, Roller E-M, Zhang T, Schüller V J, Nickels P C, Feldmann J, Liedl T (2014). Nat Nanotechnol.

[31] Zhou Y, Yang M, Sun K, Tang Z, Kotov N A (2010). J Am Chem Soc.

[32] Chen W, Bian A, Agarwal A, Liu L, Shen H, Wang L, Xu C, Kotov N A (2009). Nano Lett.

[33] Shen X, Asenjo-Garcia A, Liu Q, Jiang Q, García de Abajo F J, Liu N, Ding B (2013). Nano Lett.

[34] Fan Z, Govorov A O (2010). Nano Lett.

[35] Nemiroski A, Gonidec M, Fox J M, Jean-Remy P, Turnage E, Whitesides G M (2014). ACS Nano.

[36] Chang Y-C, Lu S-C, Chung H-C, Wang S-M, Tsai T-D, Guo T-F (2013). Sci Rep.

[37] Cataldo S, Zhao J, Neubrech F, Frank B, Zhang C, Braun P V, Giessen H (2012). ACS Nano.

[38] Oates T W H, Shaykhutdinov T, Wagner T, Furchner A, Hinrichs K (2014). Adv Mater.

[39] Tsao Y-C, Søndergaard T, Skovsen E, Gurevich L, Pedersen K, Pedersen T G (2013). Opt Express.

[40] Tsao Y-C, Fisker C, Pedersen T G (2014). Opt Express.

[41] Li Y, Su L, Shou C, Yu C, Deng J, Fang Y (2013). Sci Rep.

[42] Luo L, Chatzakis I, Wang J, Niesler F B P, Wegener M, Koschny T, Soukoulis C M (2014). Nat Commun.

[43] Choi J-H, Cho M (2014). J Phys Chem B.

[44] Liu X, Kim C N, Yang J, Jemmerson R, Wang X (1996). Cell.

[45] Liu H, Yamamoto H, Wei J, Waldeck D H (2003). Langmuir.

[46] Shervedani R K, Farahbakhsh A, Bagherzadeh M (2007). Anal Chim Acta.

[47] Wirde M, Gelius U, Nyholm L (1999). Langmuir.

[48] Shervedani R K, Bagherzadeh M, Mozaffari S A (2006). Sens Actuators, B.

[49] Lin S, Jiang X, Wang L, Li G, Guo L (2012). J Phys Chem C.

[50] Zhou Y, Nagaoka T, Zhu G (1999). Biophys Chem.

